# Transcriptome analysis reveals association of carotenoid metabolism pathway with fruit color in melon

**DOI:** 10.1038/s41598-023-31432-y

**Published:** 2023-03-27

**Authors:** Qiannan Diao, Shoubo Tian, Yanyan Cao, Dongwei Yao, Hongwei Fan, Yongping Zhang

**Affiliations:** 1 Shanghai Academy of Agricultural Sciences, Horticultural Research Institute and Shanghai Key Lab of Protected Horticultural Technology, 1018, Jinqi Road, Shanghai, People’s Republic of China; 2 Shanghai Agriculture Technology Extension and Service Center, Shanghai, 201103 People’s Republic of China

**Keywords:** Plant physiology, Transcriptomics

## Abstract

Flesh color is an important quality of melon (*Cucumis melo* L.) and is determined mainly by carotenoid content, awarding them with colors, aromas, and nutrients. enhancing the nutritional and health benefits of fruits and vegetables for humans. In this study, we performed transcriptomic analysis of two melon inbred line “B-14” (orange-flesh) and “B-6” (white-flesh) at three developmental stages. We observed that the β-carotene content of inbred line “B-6” (14.232 μg/g) was significantly lower than that of inbred line “B-14” (0.534 μg/g). RNA-sequencing and quantitative reverse transcription PCR analyses were performed to identify differentially expressed genes (DEGs) between the two inbred lines at different stages; the DEGs were analyzed using the Gene Ontology (GO) and Kyoto Encyclopedia of Genes and Genomes databases (KEGG). We identified 33 structural DEGs in different developmental periods of the two lines that were related to carotenoid metabolism. Among them, *PSY*, *Z-ISO*, *ZDS*, *CRTISO*, *CCD4*, *VDE1*, and *NCED2* were highly correlated with carotenoid content. Thus, this study provides a basis for molecular mechanism of carotenoid biosynthesis and flesh color in melon fruit.

## Introduction

Melon (*Cucumis melo* L.), an economically important fruit crop, is widely cultivated in the world. It is widely consumed because of the appearance, taste, texture, and flavor of the fruit; moreover, it is rich in vitamins, carbohydrates, carotenoids, and folic acid^1^. Carotenoids and chlorophyll (Chl) are the major pigments that determine the flesh color of melon cultivars. Orange-flesh melon is rich in carotenoids, primarily β-carotene^2^.

Carotenoids are important for maintaining human health, because they provide vitamin A, which plays a role in vision protection, antioxidation, and prevention of various cancers and cardiovascular diseases^3–5^. In plants, carotenoids participate in photosynthesis and photoprotection^6^, developmental, environmental and signaling pathways, stress response^7^, and biosynthesis of abscisic acids (ABA) and strigolactones^8^.

The carotenoid biosynthesis pathway in plants is elucidated earlier^9^: the carotenoid biosynthesis precursor geranylgeranyl diphosphate (GGPP) come from the methylerythritol phosphate (MEP) pathway. The carotenoid biosynthesis started with the condensation of two GGPP by phytoene synthase (PSY) to form colorless 15-*cis*-phytoene. Then, phytoene is subjected to a series of desaturation and isomerization reactions catalyzed by phytoene desaturase (PDS), z-carotene desaturase (ZDS), ξ-carotene desaturase (Z-ISO), and carotenoid isomerase (CRTISO), thus forming all-*trans*-lycopene. Next, β-carotene and α-carotene are synthesized by lycopene β-cyclase (LCYB) or lycopene ε-cyclase (LCYE), and then zeaxanthin and lutein are produced by the hydroxylation of β-ring carotene hydroxylase (CHYB) and cytochrome P450 type hydroxylase (CYP97A and CYP97C). Zeaxanthin cyclooxygenase (ZEP) and pansy xanthine decyclooxygenase (VDE) catalyze the synthesis of antherxanthin, violaxanthin, and neoxanthin. Carotenoids degradation involves carotenoid cleavage dioxygenases (CCDs), and 9-cis-epoxycarotenoid dioxygenases (NCEDs)^10^. Carotenoid accumulation vary greatly among species and even within the same species^11^. For example, in red tomato, watermelon, carrot, and orange cauliflower primarily accumulate lycopene and β-carotene^12–15^.

The variations observed in carotenoid content and composition in various plants are mainly related to the evolution and mutation of the carotenoid biosynthesis gene families. For example, in tomato fruit, overexpression of *ChPSY* increases carotenoid levels^16^. In red carrot, a single amino acid insertion in *LCYB2* interferes with carotenoid biosynthesis^17^. *CCD4*, a gene responsible for the loss of flesh color in white peach^18^. Variations in the promoter and coding regions of *ZEP* affect the accumulation of carotenoids in *Arabidopsis* seeds^19^.

In addition to structural genes, transcription factors (TFs), such as WRKY^20,21^, NAC^22^, MYB^23,24^, MAD^25–27^, bHLH^28,29^, and B-box (BBX) zinc-finger^26^ are involved in carotenoid metabolism in different plant species. In citrus plants, *CsERF061*, induced by ethylene, regulates carotenoid accumulation by directly activating the expression of *LCYB2* and other key carotenoid pathway genes^30^. In oriental melon, *CmWRKY49* and *CmNAC34* participate in carotenoid biosynthesis by activating the expression of *CmPSY1* and *CmLCYB*^31^.

However, how carotenoid accumulation in melon is regulated is unclear, and the detailed molecular mechanism underlying flesh color formation in different color melon have not elucidated. In our previous studies, we found that the carotenoid content in melon fruit was closely related to the flesh color (the results were not published). In addition, the orange gene of melon was initially located in the range of 3.5 Mb of chromosome 9 (the results were not published). In this study, transcriptomic analyses were conducted in two melon fruits at different developmental stages. Moreover, we investigated their phenotypes and β-carotene and Chl contents. The present study sheds light on the molecular mechanism underlying flesh-color transformation in melon fruit and will lay the foundation for carotenoid biosynthesis and fruit color formation of melon. The results will also help melon-breeding programs for improving melon quality.

## Materials and methods

### Plant material

**Figure 1:**
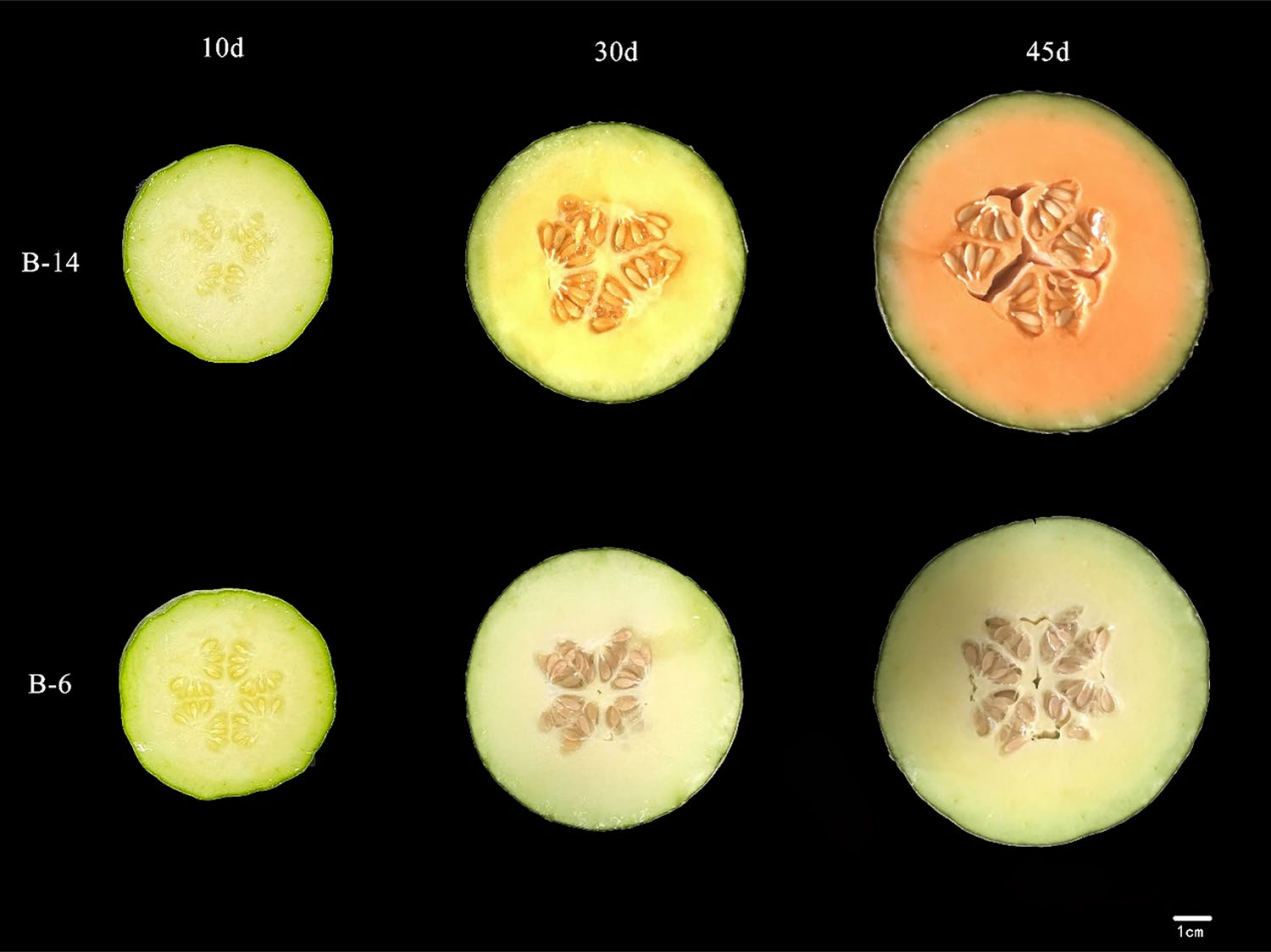
The flesh color of the two melon genotypes (“B-6” and “B-14”) at 10, 30, and 45 d after pollination (DAP). Scale bars = 1 cm.

In this study, we used two high-generation inbred melon lines “B-6” and “B-14,” which were developed by our research group. The color of the “B-6” fruits gradually change from green to white during the development period and that of “B-14” fruits changes from green to orange during the ripening period (Fig. 1). The fruits were grown in the greenhouse of Shanghai Academy of Agricultural Sciences Experiment Base during the spring season of 2021. Flowers were hand pollinated, and fruits were harvested from different plants at 10, 30, and at maturity stage (45 days) after pollination (DAP). The fruits were immediately frozen in liquid nitrogen and stored at − 80 °C for subsequent experiments. Fruit flesh were collected from three individual plants (only one fruit per individual plant was pollinated), and three biological replicates were performed for each stage. All plant studies involving (*Cucumis melo* L.) were carried out in accordance with relevant institutional, national, and international guidelines and legislation.

### Total carotenoid (Car) and β-carotene content

We collected 3 g flesh of the two melon lines at 10, 30, and 45 DAP to determine the contents of Car and β-carotene. The average concentration of total Car present in the melon pulp was determined by UV–visible absorption spectrophotometry as per the method described by Medeiros et al.^32^, using the chromatic equation reported by Biehler et al.^33^. β-Carotene content was determined by high-performance liquid chromatography (HPLC) (Waters 1525 HPLC, Waters Corp., Milford, MA, USA) , according to the method described by Medeiros et al.^32^.

### Chl content

Fruit flesh Samples (1.0 g) were mixed with 15 mL of 96% ethanol and kept in the dark for 24 h at room temperature. Next, the samples were centrifuged at 3000 × g for 10 min, and the supernatants were transferred into a cuvette with a 1‐cm optical path; 96% ethanol was used as a blank control. Absorption was measured at 665, 649, and 470 nm, with three technical replicates. The concentrations of different pigments were calculated by Wellburn and Lichtenthaler^34^.

### RNA extraction and sequencing analysis

Total RNA was extracted with three biological replicates from “B-6” and “B-14” fruits at 10, 30, 45 DAP stage using mirVana miRNA Isolation Kit (*Ambion*, Austin, TX, USA). After total RNA was extracted from the samples, RNA integrity was evaluated using an Agilent 2100 Bioanalyzer (Agilent Technologies, Santa Clara, CA, USA). Samples with RNA Integrity Number (RIN) ≥ 7 were subjected to subsequent analysis. The libraries were constructed using Kit (Illumina, San Diego, CA, USA). The RNA-seq libraries were sequenced on the Illumina sequencing platform (HiSeq™ 2500 or Illumina HiSeq X Ten), and 150-bp paired-end reads were produced.

### Bioinformatic analysis

Raw data were processed using Trimmomatic (Bolger et al. 2014)^35^. Low-quality reads and reads containing poly-N were removed to obtain clean reads. The clean reads were compared with the melon reference genome (www.melonomics.net/melonomics.html#/download) using HISAT2 (Kim et al. 2015)^36^. Gene expression levels were calculated in fragments per kilobase of transcript per million mapped reads (FKPM) for each sample^37^. Differentially expressed genes (DEGs) were identified using the DESeq R package^38^. *P* value ≤ 0.05 and fold-change ≥ 2 or fold-change ≤ 0.5 was considered as the threshold for DEGs. Hierarchical cluster analysis was applied to evaluate the gene expression pattern^39^. Gene Ontology (GO) and Kyoto Encyclopedia of Genes and Genomes (KEGG)^40^ term enrichment analysis of DEGs were carried out using R-based on the hypergeometric distribution^41^.

### Quantitative real-time PCR (RT-qPCR) analysis

RT-qPCR was performed using a LightCycler® 480 II Real-time PCR Instrument (Roche, Switzerland). The primer sequences were designed using the mRNA sequences obtained from the NCBI database and synthesized by TsingKe Biotech (Table S1). The expression levels of mRNAs were normalized to that of *18S rRNA* and were calculated using the 2^−ΔΔCt^ method^42^.

### Statistical analysis

The data were analyzed by Duncan’s multiple range tests using the SPSS package (ver. 11, SPSS, Chicago, IL, USA). Statistical significance of the data is indicated by different letters at *P* < 0.05. All data are presented as the mean ± standard deviation (SD).

### Ethical approval

The conducted experiments comply with the laws of China.

## Results

### Pigment content analysis of “B-6” and “B-14” melon cultivars

**Figure 2:**
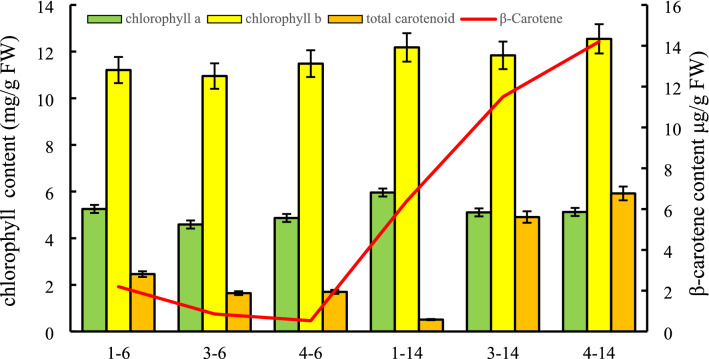
Chlorophyll a (Chl a), chlorophyll b (Chl b), total carotenoid, and β-carotene contents of the two melon cultivars at 10, 30, and 45 d after pollination (DAP); 1-6, 3-6, 4-6, and 1-14, 3-14, 4-14 represent the “B-6” and “B-14” cultivars at 10, 30, and 45 DAP, respectively.

The flesh color of “B-14” changed from yellow to orange at different developmental stages, whereas “B-6” did not exhibit any major color changes (Fig. 1). We observed that the Car and β-carotene contents of the “B-14” fruits were significantly higher than those of the “B-6” fruits at 30 and 45 DAP (Fig. 2). Chl a and Chl b contents were similar throughout the experiment between the two genotypes. In “B-14” fruits, Car and β-carotene contents gradually increased and were maximal at 45 DAP.

### Transcriptome sequencing and DEG analysis

To elucidate the regulatory mechanism behind change in melon-flesh color at the molecular level, we analyzed the transcriptomes of the two inbred line using RNA-Seq. We obtained 127.95 Gb of clean data, with approximately 44% G + C content and 94% of the mean proportion of bases with mass no less than 30 after filtration (Q30) percentage bases. Among the clean reads, 97.42–97.82% of the total mapped reads, 95.88–96.42% of the reads only matched a unique position of the reference sequence, whereas 1.25–1.57% of the reads mapped on multiple positions (Table S2). In subsequent text, G1 represents 10 DAP; G3 represents 30 DAP; G4 represents 45 DAP; “6” represents “B-6”; “14” represents “B-14”; and 1, 2, and 3 at the end of sample name represent three independent biological replicates.

**Figure 3:**
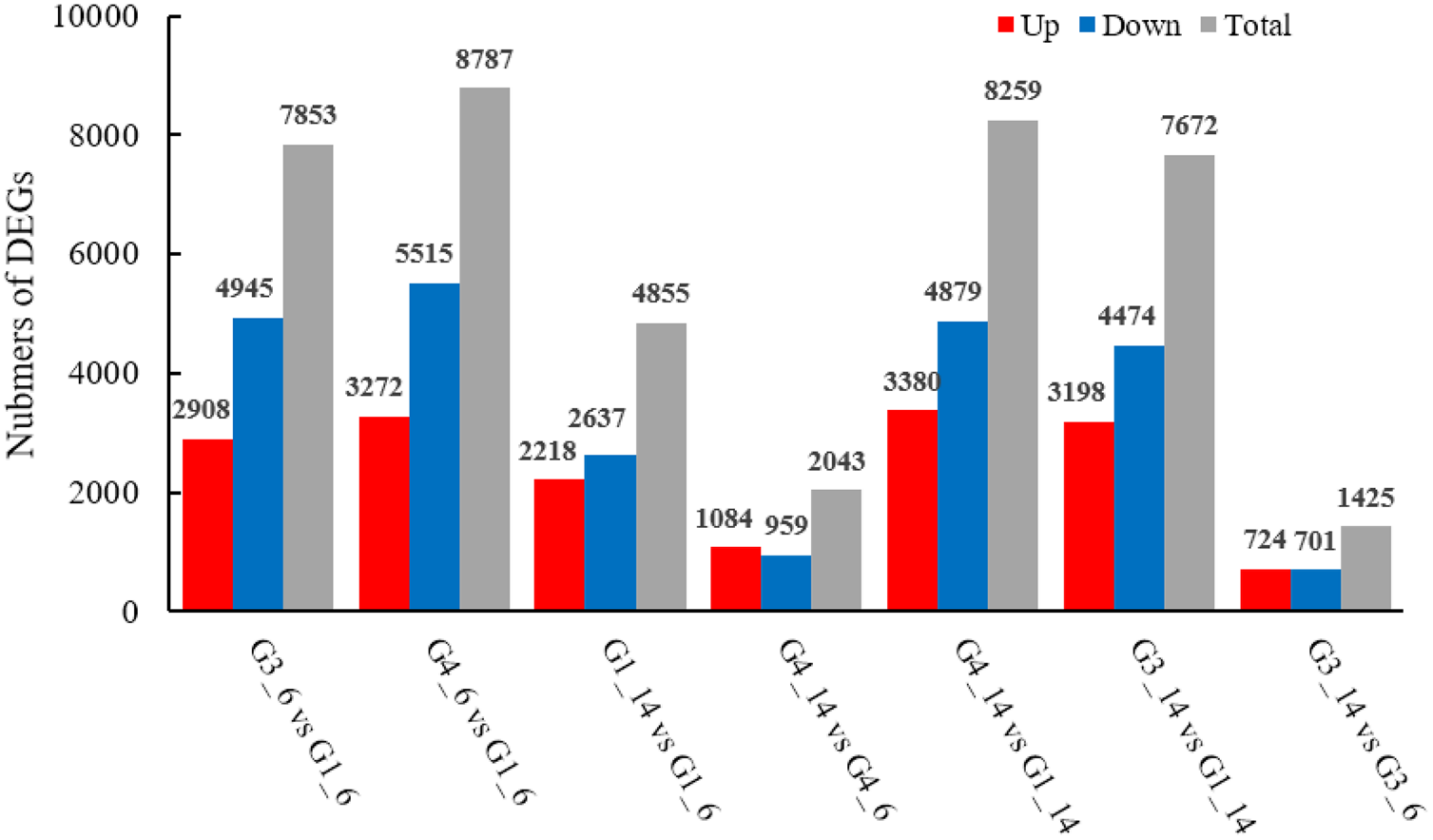
Differentially expressed genes (DEGs) between different samples of the two melon cultivars.

All clean reads were aligned to the reference genome sequence to determine the changes in the transcript or gene expression. We normalized gene expression using the FKPM value. We identified 7853 (2908 upregulated and 4945 downregulated) in “3_6” versus “1_6”, 8787 (3272 upregulated and 5515 downregulated) in “4_6” versus “1_6”, 4855 (2218 upregulated and 2637 downregulated) in “1_14” versus “4_6”, 2043 (1084 upregulated and 959 downregulated) in “4_14” versus “4_6”, 8259 (3380 upregulated and 4879 downregulated) in “4_14” versus “1_14”, 7672 (3198 upregulated and 4,474 downregulated) in “3_14” versus “1_14”, and 1425 (724 upregulated and 701 downregulated) DEGs in “3_14” versus “3_6” comparison groups, respectively (Fig. 3).

### GO and KEGG pathway functional enrichment analyses of DEGs

**Figure 4:**
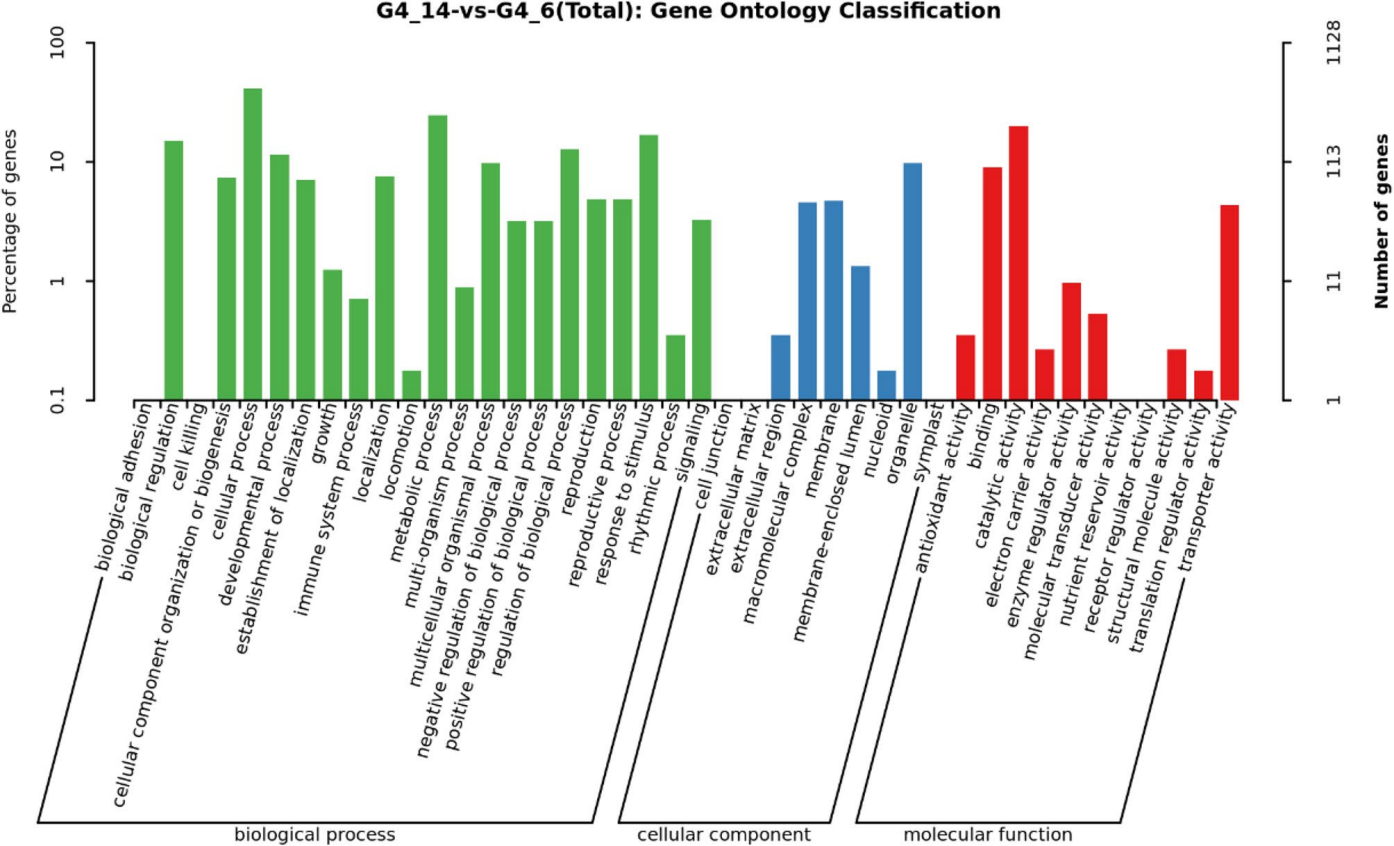
Gene Ontology (GO) enrichment analysis of different samples: The abscissa is the GO classification, the left side of the ordinate is the percentage of genes, and the right side is the gene number.

To investigate the functions of the DEGs, GO and KEGG databases were used for functional annotation. GO functional enrichment analysis found 35 GO terms were significantly enriched, including 6 for “cellular component,” 9 for “molecular function,” and 20 for “biological process.” The main “biological process” subcategories were “cellular process”, “metabolic process” and “biological regulation”; “organelle,” “macromolecular,” and “membrane” were the dominant “cellular component” subcategories; and “catalytic activity” and “binding” were the most enriched “molecular function” subcategories (Fig. 4).

**Figure 5:**
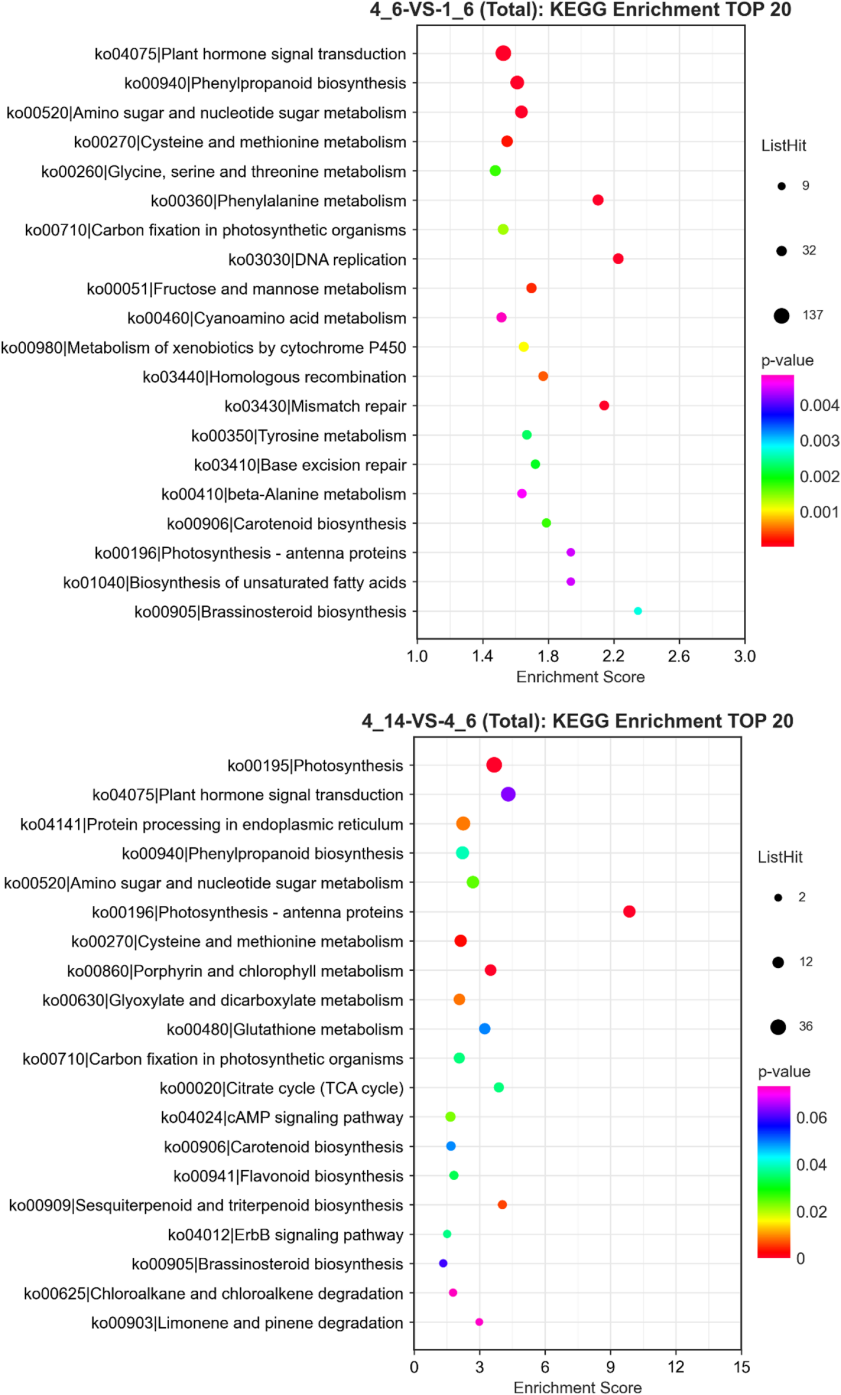
Kyoto Encyclopedia of Genes and Genomes (KEGG) enrichment analysis of different treatments. The larger the bubble, the greater the number of differential protein-coding genes contained in the entry. The bubble color changes from purple to blue to green to red. The smaller the enrichment* P* value, the more significant greater the degree.

To further elucidate the biological function of the DEGs, we analyzed them using the KEGG database. In the “4_14” versus “4_6” group, the most significantly enriched KEGG pathway was “photosynthesis-antenna proteins,” followed by “photosynthesis” and “porphyrin and chlorophyll metabolism” (Fig. 5). In the different groups, most DEGs were enriched in three pathways, including “amino sugar and nucleotide sugar metabolism,” “phenylpropanoid biosynthesis,” and “plant hormone signal transduction”. Moreover, we observed significantly enriched top 20 pathways include carotenoid biosynthesis (ko00906) in the “4_6” versus “4_14” and “4_6” versus 1_6” groups (Fig. 5).

### Analysis of DEGs associated with carotenoid metabolism

**Figure 6:**
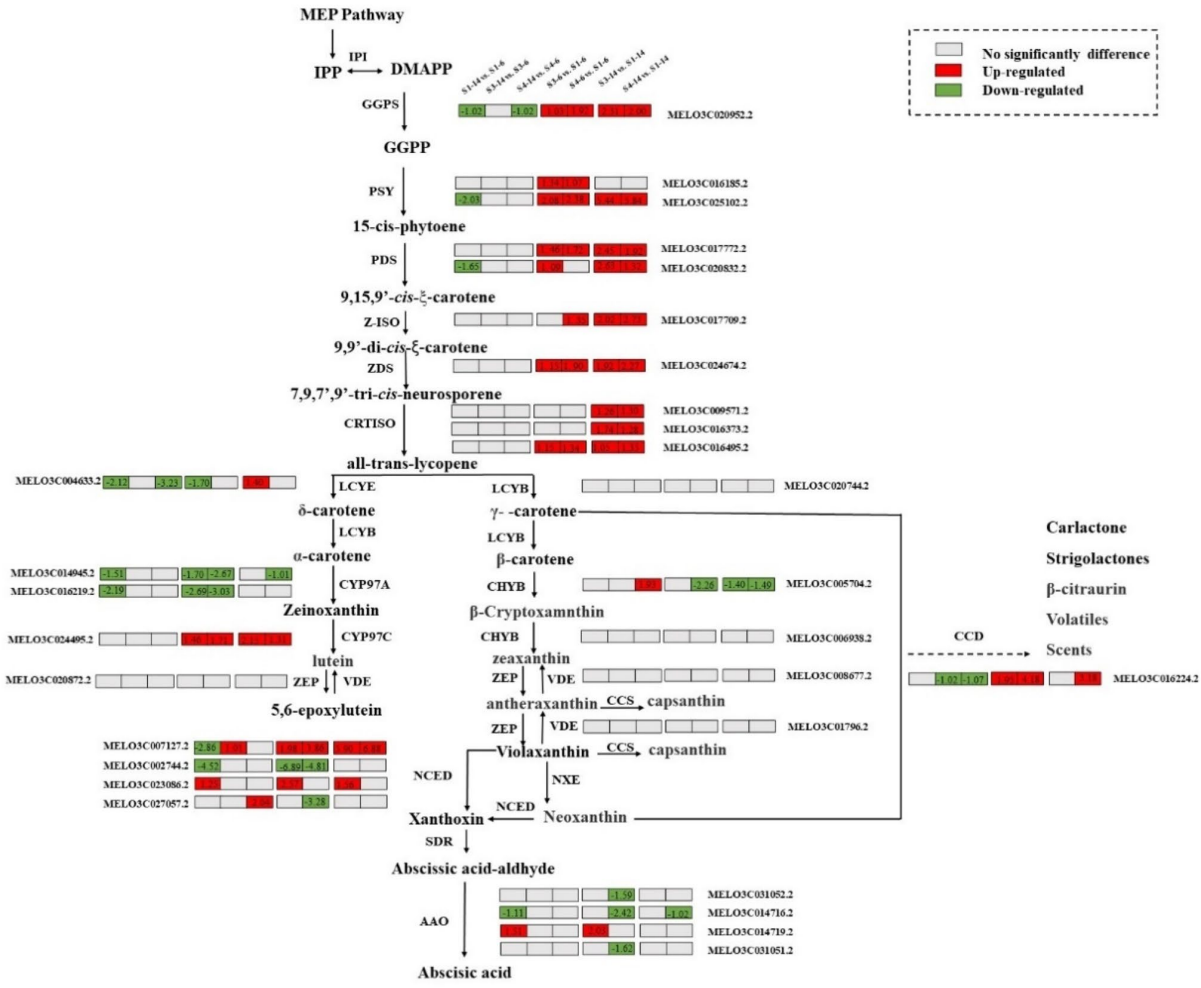
Expression patterns of genes involved in carotenoid metabolism in “1_14” versus “1_6”, “3_14” versus “3_6”, “4_14” versus “4_6”, “3_6” versus “1_6”, “4_6” versus “1_6”, “3_14” versus “1_14”, “4_14” versus “1_14” groups. Gray represents no significant difference in the comparison groups, red represents upregulated expression in the comparison groups, and green represents downregulated expression in the comparison groups.

Based on the change in fruit color and increased carotenoid content during ripening, we investigated the changes in the expression of DEGs related to the carotenoid metabolism pathway (Fig. 6). The annotation and fold change of these DEGs are summarized in Table S6. The results revealed that 33 genes were involved in the biosynthesis and metabolism of carotenoids, such as *PSY*, *PDS*, *Z-ISO*, *ZDS*, *CRTISO*, and *LCYB* (Fig. 6, Table S6). Expression of *GGPS*, *PSY*, *PDS*, CRTISO, *LCY1* and *CYP97C1* were upregulated, *CYP711A1* and *CCS* were downregulated based on the comparison of “4_14” versus “1_14”, 3_14” versus “1_14”, “4_6” versus “1_6”, 3_6″ versus “1_6” group (Fig. 6, Table. S6).

### Analysis of DEGs involved in porphyrin and Chl metabolism

**Figure 7:**
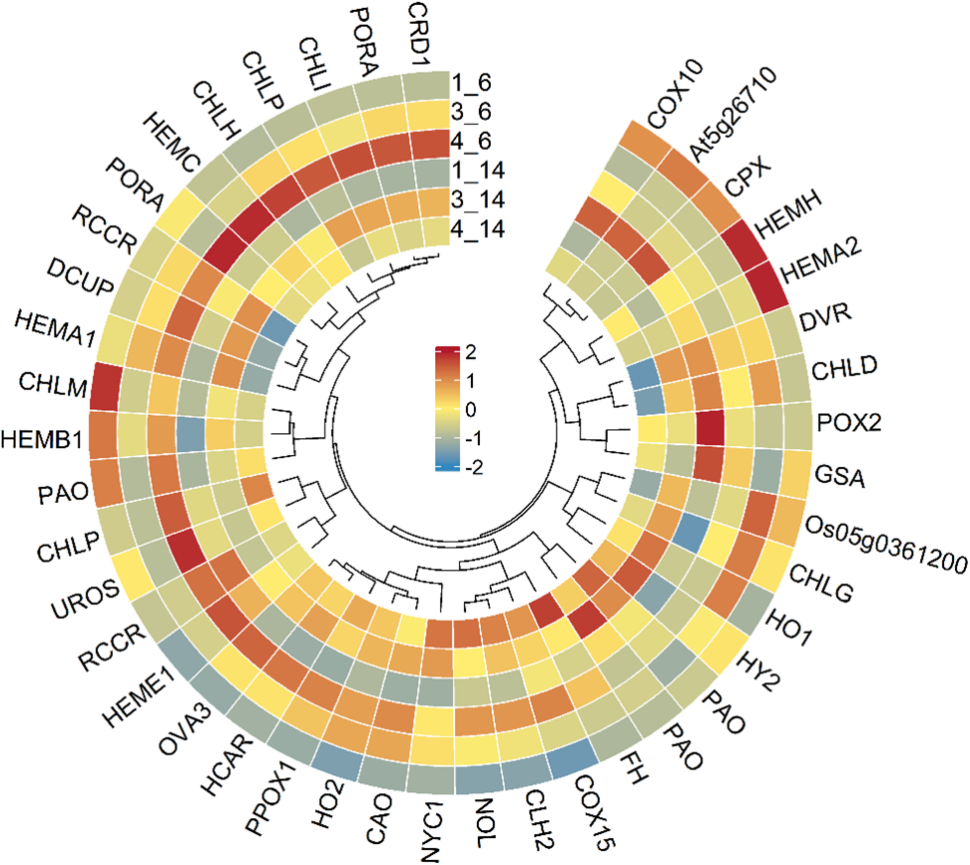
Heat map representation and hierarchical clustering of structural genes involved in porphyrin and chlorophyll metabolism in “B-14” and “B-6” melon cultivars at different growth stages.

Transcript enrichment was found to be associated with porphyrin and Chl metabolism. We identified 42 DEGs associated with porphyrin and Chl metabolism (Fig. 7, Table S7), and the expression levels of *CHLP/H*, *HemA*, *HemE*, *CRD*, and *POR* were higher in the “1_6” versus “4_6” group (Fig. 7). In the “1_6” versus 4_6” and “1_14” versus “4_14″ groups, the expression of genes encoding protoporphyrinogen oxidase 1 (*POX1*), geranylgeranyl diphosphate reductase (*CHLP*), magnesium-chelatase subunit (*ChlH*), 7-hydroxymethyl chlorophyll a reductase (*HCAR*), magnesium-protoporphyrin IX monomethyl ester [oxidative] cyclase (*CRD1*), and probable chlorophyll(ide) b reductase (*NYC1*) was upregulated (Table S7).

### Differentially expressed TFs

Among the differentially expressed TFs, six MYB TFs, three ethylene-responsive TFs (ERF), five bHLH TFs, seven NAC domain-containing proteins, and eight WRKY TFs were significantly upregulated in the “4_14” versus “4_6” group. Moreover, BBX zinc finger protein, zinc finger protein, basic leucine zipper, heat shock factor protein, and MADS-box TF were also significantly upregulated in the “4_14” versus 4_6” group.*MYB48, ERF039, ERFLEP, bHLH171, BBX18, NAC73, NAC21/22,* and *MADS13* were upregulated, *MYB63, ERF106,061,* Basic leucine zipper 43, NAC domain-containing protein 2,55,92, *WRKY24,*75, *HSF24,* ERF *CRF6* were downregulated in the “4_14” versus “1_14” and “3_14” versus “1_14” groups (Table 1).

**Table 1:** Differential expression analysis of transcription factors.

Gene ID	Description	log2 Fold change		
		4_14 versus 1_14	3_14 versus 1_14	4_14 versus 4_6
MELO3C016789.2	Transcription factor *MYB73*	–	–	1.2998
MELO3C020701.2	Transcription factor *MYB48*	–	1.5287	1.2545
MELO3C024440.2	Transcription factor *MYBS3*	–	–	1.0868
MELO3C034877.2	Transcription factor *MYB63*	− 2.9518	− 3.3428	3.4374
MELO3C034933.2	Transcription factor *MYB12*	1.9336	–	2.2068
MELO3C034933.2	Transcription factor* MYB12*	1.9336	–	2.2068
MELO3C005466.2	Ethylene-responsive transcription factor *ERF106*	− 4.0699	− 5.0526	1.3450
MELO3C005747.2	Ethylene-responsive transcription factor *ERF061*	− 5.2953	− 4.8672	2.0750
MELO3C006430.2	Ethylene-responsive transcription factor 1B	− 3.9087	–	2.5257
MELO3C013926.2	Ethylene-responsive transcription factor *ERF039*	1.5905	3.9636	− 1.5315
MELO3C002608.2	Transcription factor *bHLH162*	1.7361	− 3.4999	1.1336
MELO3C003421.2	Transcription factor *bHLH71*	− 2.4912	2.8717	3.7333
MELO3C007341.2	Transcription factor *bHLH112*	–	–	1.5046
MELO3C014408.2	Transcription factor *bHLH69*	− 1.6732	–	3.2872
MELO3C015253.2	Transcription factor *bHLH149*	− 1.3051	–	1.0149
MELO3C023131.2	Magnesium-chelatase subunit* ChlH*	5.4357	5.2853	− 1.3508
MELO3C017424.2	Transcription factor *bHLH35*	1.4022	–	2.0419
MELO3C007886.2	B-box zinc finger protein 18 *BBX18*	3.1121	2.8876	1.6896
MELO3C020703.2	Zinc finger protein *ZAT11*	1.4957	–	3.2557
MELO3C005173.2	Basic leucine zipper 43	− 1.3813	− 1.4699	2.6018
MELO3C007865.2	bZIP transcription factor 53	2.7069	–	1.5434
MELO3C010788.2	Basic leucine zipper 4	3.8796	1.4713	2.3542
MELO3C019312.2	Basic leucine zipper 34	/	–	4.7436
MELO3C002628.2	NAC domain-containing protein 73	4.6973	1.9801	1.1275
MELO3C007255.2	NAC domain-containing protein 10	5.9607	–	6.9270
MELO3C010555.2	NAC domain-containing protein 21/22	1.4082	2.0164	1.7337
MELO3C010632.2	NAC domain-containing protein 2	− 3.9768	− 4.3946	1.1002
MELO3C016536.2	NAC domain-containing protein 55	− 1.2900	− 2.0017	1.4025
MELO3C022002.2	NAC domain-containing protein 92	− 1.4771	− 2.5148	1.3557
MELO3C027409.2	NAC domain-containing protein 43	–	–	3.2147
MELO3C008175.2	Probable WRKY transcription factor 75	4.6866	–	5.8101
MELO3C009097.2	WRKY DNA-binding transcription factor 70	–	–	2.7393
MELO3C009127.2	WRKY transcription factor *WRKY24*	− 3.0631	− 2.4047	1.5240
MELO3C014896.2	Probable WRKY transcription factor 75	4.6847	–	1.5008
MELO3C017743.2	Probable WRKY transcription factor 75	− 2.7759	− 10.1545	3.5068
MELO3C020489.2	WRKY transcription factor WRKY24	− 1.5920	–	2.0956
MELO3C020963.2	Probable WRKY transcription factor 51	–	–	2.2669
MELO3C030287.2	Probable WRKY transcription factor 50	–	–	3.3773
MELO3C007560.2	Heat shock factor protein *HSF24*	− 1.5324	− 5.1024	4.9703
MELO3C011671.2	Ethylene-responsive transcription factor *CRF6*	− 2.6952	− 5.4191	5.1621
MELO3C033521.2	MADS-box transcription factor 13 *MADS13*	1.5089	1.4745	− 1.0050
MELO3C005088.2	Ethylene-responsive transcription factor *LEP*	6.5761	6.6288	–

### Validation of gene expression levels by RT-qPCR

**Figure 8:**
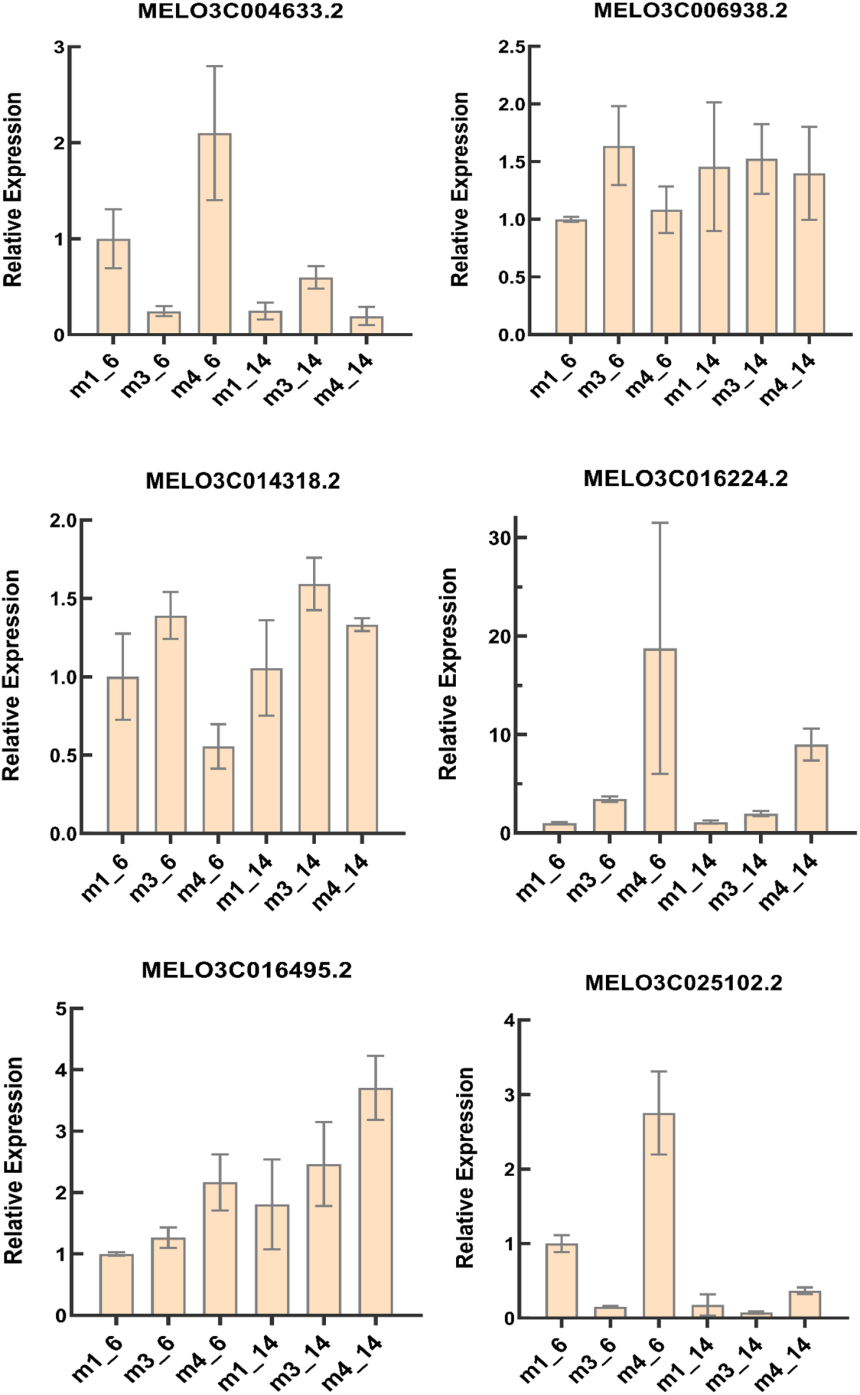
The expression levels of six carotenoid-related genes in flesh of “B-14” and “B-6” melon cultivars at different developmental stages.

To validate the results of RNA-Seq, we randomly selected six genes for RT-qPCR analysis, including six genes related to carotenoid metabolism (Fig. 8). The expression patterns obtained by RT-qPCR were similar to those obtained by RNA-Seq, demonstrating the accuracy of the RNA-Seq results.

## Discussion

The yellow, orange, and red phenotype of plants is associated with carotenoid content^43^. The composition and content of carotenoids vary between different varieties of plants. For example, red tomato and watermelon are rich in lycopene^44,45^. Carrot and melon are abundant in β-carotene^14,46^. Some leafy vegetables, such as broccoli and kale, contain large amounts of lutein, β-carotene, and zeaxanthin^47,48^. In the current study, the fruit-flesh color of melon cultivars “B-14” and “B-6” was white-green at the early developmental stage, and then changed to orange in the “B-14” cultivar and to white in the “B-6” cultivar at the mature stage (Fig. 1). Similarly, the contents of Car and β-carotene in the “B-14” fruits were significantly higher than those in the “B-6” fruits, indicating that the orange flesh-color in melon is due to β-carotene accumulation, whereas the white flesh-color of “B-6” was mainly due to the lack of carotenoids (Fig. 2). These results were similar to those observed in watermelon^21^, cucumber^49^, squash^50^.

Carotenoid accumulation is regulated by transcriptional regulation of carotenoid metabolic pathway genes^51^. We identified and analyzed 33 carotenoid pathway genes in this study and found the expression levels of upstream synthesis genes, *PSY* (MELO3C025102.2), *Z-ISO* (MELO3C017709.2), *CRTISO* (MELO3C009571.2, MELO3C016495.2), and *ZDS* (MELO3C024674.2), and downstream synthesis genes, *CCD4* (MELO3C016224.2), *NCED2* (MELO3C007127.2), and *VDE1* (MELO3C008677.2), were the highest in the “4_14” group, followed by those in the “3_14” and “1_14” groups (Fig. S1, Table S6). Furthermore, seven upregulated and three downregulated genes were observed in the “4_14” group compared with the “1_14” group.

As shown in Fig. 6, *PSY* is involved in the key steps of the carotenoid metabolism pathway^52^. A study reported that overexpression of *PSY1* in tomato plants significantly increased the carotenoid content in tomato fruit^53^. In our study, *CmPSY* was highly expressed at the mature stage, revealing that *PSY* plays an essential role in carotenoid accumulation in melon. Besides, the downregulation of *CmCHYB* partially contributed to the accumulation of β-carotene. Therefore, the increased synthesis of β-carotene in orange-flesh melon “B-14” at the mature stage could be due to upregulation of biosynthesis genes *CmPSY*, *CmPDS*, *CmZDS*, *CmZ-ISO*, and *CmCRTISO*. The downregulation of *CmCHYB* prevented the flux into downstream products, whereas upregulation of *CYP97C* promoted the conversion of zeaxanthin into lutein. Meanwhile, the upregulation of degradation genes, *CmNCED* and *CmCCD4*, could have decreased the total carotenoid content as well as the content of individual carotenoids.

In this study, we list the top 20 significantly DEGs (up-regulated and down-regulated) of each comparison were shown in supplemental file (Table S3, S4, S5). We found that the expression of *WRKY75* was significantly upregulated in the “4_14” versus “4_6” and “4_14” versus “1_14”. which was consistent with the fruit color of melon*.* Previous studies also found that *OfWRKY3, an O. fragrans* transcription factor, could alter the carotenoid profiles by regulating *OfCCD4* expression^20^. *SlWRKY35* positively regulates carotenoid biosynthesis by activating the MEP pathway in tomato fruit. *CmWRKY49* participated in carotenoid biosynthesis by activating the expression of *CmPSY1* in orange fleshed oriental melon^54^. So, we suspected that *WRKY75* may be a regulator for carotenoid biosynthesis accumulation in melon fruit.

Plant hormones can directly or indirectly affect the biosynthesis of carotenoid in many plants^55–58^. Auxin has been reported to retard carotenoid accumulation in tomato fruit^55^. Consistent with this, our results showed that compared with “4_14”, Xyloglucan endotransglucosylase/hydrolase protein 22 (XTH22, MELO3C017480.2) involves in response to auxin, ABC transporter B family member 11 (ABCB11, MELO3C008741.2) associated with auxin efflux transmembrane transporter activity, inactive TPR repeat-containing thioredoxin (TTL3, MELO3C010417.2) associated with auxin-activated signaling pathway, were highly expressed in “1_14”. Meanwhile, we found that the expressions of *CYP97A3* and *CHYB* genes were down-regulated in “4_14”. A recent study suggested that a loss of function in the carotene hydroxylase *CYP97A3* gene related with high α-carotene content in orange carrots^59^. We speculated that auxin might regulate the expression levels of *CYP97A3* and *CHYB* to affect carotenoid accumulation in melon fruit.

Similarly, ABA and JA are also involved in affecting carotenoid accumulation in tomato^60,61^. In this study, compared with “4_6”, an alkaline ceramidase (ACER, MELO3C006305.2) involves in abscisic acid-activated signaling pathway were highly expressed in “4_14”. On the contrary, Protein FAF-like (At5g22090, MELO3C015055.2) associated with negative regulation of abscisic acid activated signaling pathway, glutamate receptor 3.3 (GLR3.3, MELO3C017043.2) associated with jasmonic acid mediated signaling pathway were highly expressed at “4_6”. Suppression of the key ABA biosynthetic gene, *SlNCED1*, resulted in down-regulation of *SlLCY-B* expression, and up-regulation of *SlPSY1* expression with increased levels of lycopene and β-carotene^62^. However, whether ABA directly regulate carotenoid pathway gene expression in current study remains unknown.

In the present study, 42 DEGs related to porphyrin and Chl metabolism were identified (Fig. 7, Table S7). Compared with “1_6”, the expression levels of *CHLP/H*, *HemA*, *HemE*, *CRD*, and *POR* were higher in the 4_6” group (Fig. 7). In the “1_6” versus “4_6” and the “1_14” versus “4_14” groups, the expression of *POX1*, *CHLP*, *ChlH*, *HCAR*, and *CRD1* was upregulated (Table S7). However, genes related to Chl degradation, such as *NYC*, *PAO*, and *RCCR*, were highly expressed only in the “4_6” and “4_14” groups, which may directly lead to rapid degradation of Chl, resulting in etiolation and bleaching^63^.

Many TFs regulate carotenoid metabolism and influence carotenoid content. For example, SlMYB72, SlWRKYs, and SlBBX20 were found to bind to *SlPSY* and regulate the synthesis of carotenoids in tomato^25,63,64^. In this study, two *MYB12*, *BBX18*, and two *WRKY75*, were upregulated in the “4_6” versus “4_14” and “1_14” versus “4_14” groups (Table 1). Moreover, several MADS-box regulators influence carotenoid biosynthesis. *CsMADS5* and *CsMADS6* influence carotenoid metabolism by upregulating *LCYB1* and other carotenogenic genes in citrus fruits^24,26^. Recently, NAC TFs that participate in carotenoid metabolism have also been reported in papaya, apple, and tomato^65–67^. Our results revealed that NAC domain-containing protein 21/22 was upregulated in all treatments in both inbred lines (Table 1), suggesting that these TFs may be related to carotenoid metabolism.

## Conclusion

In this study, we found that carotenoid content gradually increased in the flesh of “B-14” cultivar and the color changed from green to orange. Transcriptome analysis revealed that *PSY*, *PDS*, *ZDS*, *Z-ISO*, *CRTISO*, and *CHYB* could play a crucial role in carotenoid accumulation; thus, they could be responsible for the orange–red flesh-color of the mature fruit. Moreover, a number of DEGs were enriched in the Chl biosynthesis pathways. Our study provides insights into the molecular mechanisms underlying changes in fruit color of melon during different developmental stages. This study was performed mainly at the transcription level, thus further in-depth analyses are required.

## Supplementary Information

Supplementary Information.

## Data Availability

Data supporting the results and conclusions are included in both the article and additional files. All the transcriptome data have been deposited in the NCBI’s Gene Expression Omnibus (GEO) and accession number: GSE220109.
