# Supplementary material for: Transcriptome analysis reveals association of carotenoid metabolism pathway with fruit color in melon
Source: Sci Rep. 2023 Mar 27;13:5004. doi: 10.1038/s41598-023-31432-y (PMC10043268; doi:10.1038/s41598-023-31432-y)
Supplement: Supplementary file 1 — Supplementary Information. [file 41598_2023_31432_MOESM1_ESM.pdf]

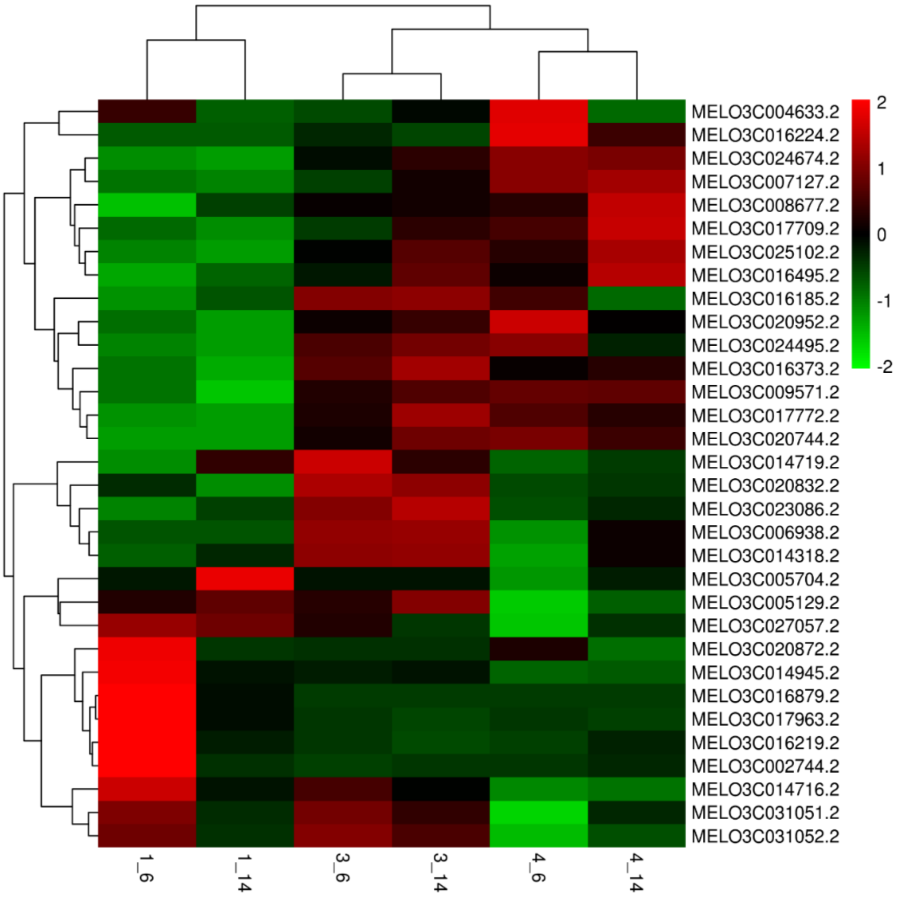

Fig. S1 Heat map representation and hierarchical clustering of structural genes involved in carotenoid biosynthesis in "B-14" and "B-6" melon cultivars at different growth stages.

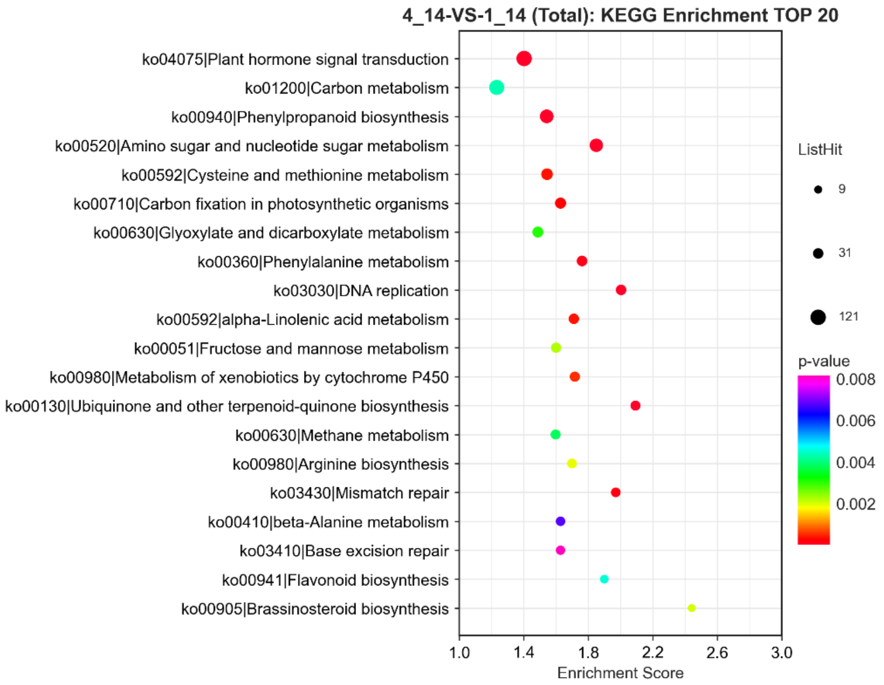

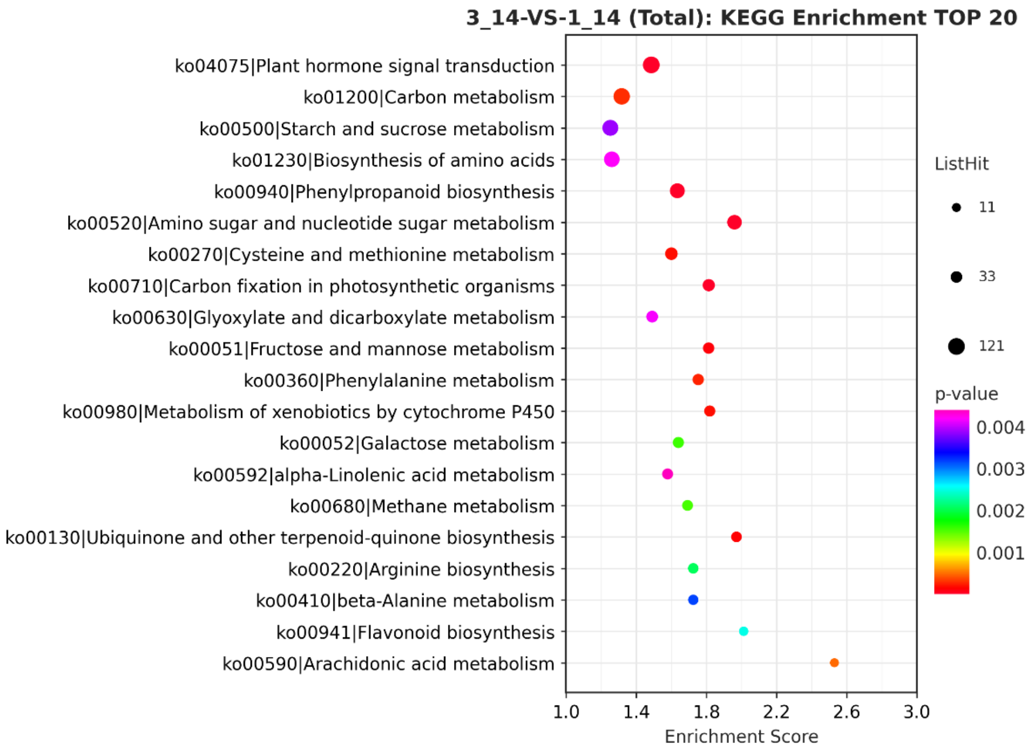

Fig. S2 Kyoto Encyclopedia of Genes and Genomes (KEGG) enrichment analysis in 4_14 VS 1_14 and 3_14 VS 1_14.

Table S1 Primer sequences of genes selected for qRT-PCR

Num	Gene Symbol	Forward primer(5->3)	Reverse primer(5->3)
1	18S-RNA	GTGATGGTGTGAGTCACACTGTTC	ACGACCAGCAAGGTCCAAAC
2	MELO3C025102.2	TCTTCCCTCGTGCGATAC	TTAAACCGCCCAAGAAATTCCA
3	MELO3C006938.2	CGGTCTTTGGGATGGCCTA	GCAGCAACCTTTCTGAAGT
4	MELO3C004633.2	GGTTATTGGTTGTGGTCCTG	TGGACCAATGAGTCCAACTT
5	MELO3C014318.2	CAAAGCCAATCCTCTCTTTGA	GACAGTGGTGAATGCAGC
6	MELO3C016495.2	GGACCTTTGCTCCAGTATG	TACTGCTGGAGCTACGTG
7	MELO3C016224.2	GCTCCAGTCGATGAGCTAC	TTTGGGCCGTTACGAATGT

Table S2 Throughout and quality of Illumina sequencing

Sample	Raw Reads	Clean Reads	Q30(%)	GC(%)	Total mapped reads	Uniquely mapped	Multiple mapped
1_14_1	7.49G	7.10G	94.92	45.44	48025427(97.64%)	47377429(96.33%)	647998(1.32%)
1_14_2	7.33G	7.01G	94.77	45.00	47051052(97.49%)	46366449(96.07%)	684603(1.42%)
1_14_3	7.45G	7.13G	94.68	44.87	47747528(97.51%)	47104966(96.19%)	642562(1.31%)
1_6_1	7.09G	6.75G	94.61	45.04	45551261(97.67%)	44968412(96.42%)	582849(1.25%)
1_6_2	7.37G	7.03G	94.50	45.01	47279279(97.59%)	46674228(96.34%)	605051(1.25%)
1_6_3	7.59G	7.20G	94.56	45.05	48628853(97.70%)	47979822(96.40%)	649031(1.30%)
3_14_1	7.51G	7.23G	94.65	44.48	48134896(97.60%)	47370941(96.06%)	763955(1.55%)
3_14_2	7.65G	7.29G	94.72	44.46	49034103(97.66%)	48270911(96.14%)	763192(1.52%)
3_14_3	7.37G	7.06G	94.68	44.26	47230695(97.66%)	46538134(96.23%)	692561(1.43%)

3_6_1	7.16G	6.86G	94.83	44.62	46052252(97.75%)	45340498(96.24%)	711754(1.51%)
3_6_2	7.10G	6.84G	94.89	44.47	45640762(97.65%)	44934361(96.13%)	706401(1.51%)
3_6_3	7.59G	7.26G	94.97	44.52	48835578(97.76%)	48080335(96.25%)	755243(1.51%)
4_14_1	7.71G	7.34G	94.58	44.83	49281632(97.42%)	48504505(95.88%)	777127(1.54%)
4_14_2	7.74G	7.41G	94.98	44.52	49723870(97.62%)	48922828(96.04%)	801042(1.57%)
4_14_3	7.23G	6.94G	94.82	44.77	46397273(97.45%)	45704575(96.00%)	692698(1.45%)
4_6_1	7.41G	7.07G	94.88	45.00	47644115(97.75%)	46921764(96.27%)	722351(1.48%)
4_6_2	7.44G	7.10G	94.85	45.00	47736904(97.58%)	47044095(96.17%)	692809(1.42%)
4_6_3	7.71G	7.33G	94.77	45.02	49511494(97.82%)	48776651(96.37%)	734843(1.45%)

Table S3 Top 20 upregulated and downregulated genes in 4_14 vs 4_6

Gene	Annotation	p-value	Regulation
MELO3C008175.2	Probable WRKY transcription factor 75	7.85E-247	Up
MELO3C006443.2	Enoyl-CoA delta isomerase 2, peroxisomal	1.19E-128	Down
MELO3C025797.2	Flavonoid 3'-monooxygenase	8.79E-90	Up
MELO3C007560.2	Heat shock factor protein HSF24	7.39E-83	Up
MELO3C005620.2	Homeobox protein HD1	5.39E-65	Up
MELO3C020676.2	Phenolic glucoside malonyltransferase 1	6.35E-62	Up
MELO3C002943.2	Beta-amyrin synthase	3.29E-56	Down
MELO3C016210.2	Pectin acetylesterase 8	3.95E-55	Up
MELO3C014477.2	Cyclic nucleotide-gated ion channel 1	6.26E-50	Down
MELO3C017959.2	DExH-box ATP-dependent RNA helicase DExH8	5.47E-49	Up
MELO3C014666.2	Dirigent protein 20	1.63E-48	Down
MELO3C005493.2	Acid phosphatase 1	5.48E-47	Down
MELO3C006305.2	Alkaline ceramidase	9.26E-47	Up
MELO3C005737.2	Light-sensor Protein kinase	1.58E-46	Up
MELO3C010254.2	Dicer-like protein 4	1.08E-44	Up
MELO3C035241.2	Somatic embryogenesis receptor kinase 2	1.92E-44	Up
MELO3C005504.2	Putative disease resistance protein At4g19050	3.83E-44	Up
MELO3C017043.2	Glutamate receptor 3.3	2.05E-43	Down
MELO3C015055.2	Protein FAF-like, chloroplastic	4.72E-43	Down
MELO3C010825.2	Putative receptor-like protein kinase At3g47110	1.79E-42	Down

Table S4 Top 20 upregulated and downregulated genes in 4_14 vs 1_14

Gene	Annotation	p-value	Regulation
MELO3C004143.2	Germin-like protein 5-1, Os05g0277500	0	Down
MELO3C007003.2	Vacuolar-sorting receptor 6, VSR6	0	Up
MELO3C008010.2	Heavy metal-associated isoprenylated plant protein 7, HIPP07	0	Up
MELO3C012099.2	Ribonuclease 1, RNS1	0	Down
MELO3C017480.2	Xyloglucan endotransglucosylase/hydrolase protein 22, XTH22	0	Down
MELO3C025347.2	Probable chlorophyll(ide) b reductase NYC1, chloroplastic	0	Up
MELO3C025448.2	Enoyl-CoA delta isomerase 1, peroxisomal, ECI1	9.49E-300	Down

MELO3C015312.2	Respiratory burst oxidase homolog protein B, RBOHB	4.91E-287	Down
MELO3C006405.2	Long-chain-alcohol oxidase FAO1	2.81E-278	Up
MELO3C003294.2	Vacuolar cation/proton exchanger 3, CAX3	3.05E-277	Up
MELO3C021479.2	AAA-ATPase ASD, mitochondrial, AATP1	8.77E-277	Down
MELO3C010681.2	Cysteine-rich receptor-like protein kinase 2, CRK2	6.97E-261	Down
MELO3C008741.2	ABC transporter B family member 11, ABCB11	3.85E-259	Down
MELO3C010417.2	Inactive TPR repeat-containing thioredoxin TTL3	4.26E-255	Down
MELO3C012305.2	Probable S-adenosylmethionine-dependent methyltransferase CRG1	5.82E-255	Down
MELO3C014857.2	Lysine histidine transporter 1, LHT1	1.65E-254	Down
MELO3C021253.2	Beta-glucosidase-like SFR2, chloroplastic	5.15E-251	Up
MELO3C008175.2	Probable WRKY transcription factor 75, WRKY75	3.49E-249	Up
MELO3C010739.2	Acyl-[acyl-carrier-protein] desaturase	1.25E-243	Up
MELO3C002729.2	Probable purine permease 10, PUP10	2.56E-237	Down

Table S5 Top 20 upregulated and downregulated genes in 3_14 vs 1_14

Gene	Annotation	p-value	Regulation
MELO3C003294.2	Vacuolar cation/proton exchanger 3	0	Up
MELO3C004143.2	Germin-like protein 5-1	0	Down
MELO3C004455.2	Umecyanin	0	Down
MELO3C007334.2	Equilibrative nucleotide transporter 3	0	Down
MELO3C009596.2	F-box/kelch-repeat protein At2g44130	0	Down
MELO3C012099.2	Ribonuclease 1	0	Down
MELO3C015552.2	Sucrose synthase	0	Down
MELO3C025264.2	Protein PIN-LIKES 6	3.37E-298	Up
MELO3C016513.2	Metallothionein-like protein type 3	1.47E-293	Up
MELO3C007576.2	Probable WRKY transcription factor 15	1.75E-286	Down
MELO3C026342.2	Calmodulin-3	8.54E-277	Down
MELO3C008413.2	Stearoyl-[acyl-carrier-protein] 9-desaturase 6, chloroplastic	3.07E-275	Down
MELO3C026755.2	Probable xyloglucan endotransglucosylase/hydrolase protein 5	9.48E-272	Down
MELO3C012305.2	Probable S-adenosylmethionine-dependent methyltransferase CRG1	2.60E-271	Down
MELO3C026279.2	Cell division cycle protein 48 homolog	2.54E-267	Up
MELO3C015312.2	Respiratory burst oxidase homolog protein B	4.63E-264	Down
MELO3C006821.2	21 kDa protein	4.33E-261	Down

Table S6 Differentially expressed genes involved in carotenoid biosynthesis

Gene	annotation	1_14 vs 1_6 3_6 vs 1_6 3_14 vs 1_14 4_6 vs 1_6 4_14 vs 1_14 4_14 vs 4_6																	
		log2Foldchange						p-value						FOR					
MELO3C020952.2	GGPS, Geranylgeranyl pyrophosphate synthase	-1.0226	1.0288	2.3124	1.9234	1.9990	-1.0167	1.21E-07	4.64E-20	2.93E-26	1.62E-28	5.13E-29	2.70E-11	7.63E-07	2.66E-19	3.00E-25	1.87E-27	5.36E-28	1.29E-09
MELO3C016185.2	PSY, Phytoene synthase	/	1.3424	/	1.0690	/	/	/	0.000225	/	0.0054	/	/	/	0.00043	/	0.009236	/	/
MELO3C025102.2	PSY, Phytoene synthase	-2.7035	2.0763	5.4397	2.3818	5.8420	/	1.40E-40	2.48E-55	8.20E-10	2.98E-26	2.39E-19	/	1.30E-38	4.49E-54	6.83E-100	3.08E-25	1.07E-19	/
MELO3C017772.2	PDS, 15-cis-phytoene desaturase	/	1.4582	2.4526	1.7187	1.9181	/	/	3.50E-31	2.68E-26	2.89E-16	6.16E-34	/	/	3.13E-30	2.75E-25	1.71E-15	7.66E-33	/
MELO3C020832.2	PDS, 15-cis-phytoene desaturase	-1.6453	1.0935	2.6289	/	1.3161	/	5.83E-10	1.80E-09	6.86E-30	/	6.83E-05	/	5.11E-09	5.54E-09	8.21E-29	/	0.000148	/
MELO3C017709.2	Z-ISO,15-cis-zeta-carotene isomerase	/	/	2.0190	/	2.7344	/	/	/	3.27E-14	/	2.18E-31	/	/	/	1.72E-13	/	2.49E-30	/
MELO3C024674.2	Zeta-carotene desaturase	/	1.1512	1.9173	/	2.2734	/	/	1.45E-33	3.49E-26	/	1.27E-49	/	/	1.40E-32	3.56E-25	/	2.67E-48	/
MELO3C009571.2	CRTISO, Prolycopene isomerase	/	/	1.2593	/	1.2993	/	/	/	7.48E-16	/	3.51E-36	/	/	/	4.38E-15	/	4.74E-35	/
MELO3C016373.2	CRTISO, Prolycopene isomerase	/	/	1.7414	/	1.2827	/	/	/	1.00E-15	/	8.62E-10	/	/	/	5.81E-15	/	2.95E-09	/
MELO3C016495.2	CRTISO, Prolycopene isomerase	/	1.1469	1.0479	1.3439	1.3500	/	/	1.52E-09	4.54E-06	3.07E-07	2.84E-12	/	/	4.72E-09	1.23E-05	8.81E-07	1.17E-11	/
MELO3C020744.2	LCY1, Lycopene beta cyclase	/	/	1.3624	1.2679	1.1679	/	/	/	3.18E-15	5.95E-19	9.16E-28	/	/	/	1.79E-14	4.13E-18	9.10E-27	/
MELO3C004633.2	LUT2, Lycopene epsilon cyclase	-2.1150	-1.6981	1.3980	/	/	-3.2319	1.42E-07	2.67E-06	0.0040	/	/	1.71E-18	8.88E-07	6.34E-06	0.007255	/	/	2.19E-16
MELO3C016224.2	CCD4, Probable carotenoid cleavage dioxygenase 4	/	1.9508	/	4.1800	3.1769	-1.0735	/	4.21E-28	/	1.45E-19	1.69E-32	0.0130	/	3.38E-27	/	1.04E-18	2.01E-31	0.05771
MELO3C014945.2	CYP97A3, Protein LUTEIN DEFICIENT 5	-1.5061	-1.704	/	-2.6731	-1.0071	/	2.22E-22	1.82E-32	/	3.03E-72	2.64E-07	/	6.39E-21	1.70E-31	/	1.60E-70	7.34E-07	/
MELO3C016219.2	CYP707A2, Abscisic acid 8'-hydroxylase	-2.1856	-2.6914	/	-3.0331	/	/	0.000659		/	1.86E-12	/	/	0.002131	8.04E-06	/	8.40E-12	/	/
MELO3C024495.2	CYP97C1, Carotene epsilon-monooxygenase	/	1.4580	2.1488	1.7063	1.3138	/	/	2.26E-22	2.67E-52	7.39E-09	1.34E-11	/	/	1.43E-21	7.19E-51	2.47E-08	5.27E-11	/
MELO3C016879.2	CYP711A1, Cytochrome P450 711A1	-2.7290	-2.6913	-4.7044	-7.5797	-4.3958	/	1.61E-31	3.41E-06	7.53E-48	1.11E-14	9.71E-19	/	8.90E-30	1.07E-13	1.75E-46	4.81E-145	6.06E-18	/

MELO3C017963.2	CCS, Capsanthin /capsorubin synthase	-2.2355	-3.3654	-1.8578	-3.3437	-1.6063	/	1.41E-12	1.29E-45	9.31E-07	1.20E-19	9.32E-06	/	1.69E-11	1.80E-44	2.71E-06	8.66E-19	2.21E-05	/
MELO3C014716.2	AAO3, Abscisic-aldehyde oxidase	-1.1088	/	/	-2.4165	-1.0171	/	5.61E-12	/	/	1.74E-48	5.19E-10		6.21E-11	/	/	4.47E-47	1.81E-09	/
MELO3C014719.2	AAO2, Indole-3-acetaldehyde oxidase	1.5136	2.0277	/	/	/	/	3.09E-10	1.67E-25	/	/	/		2.79E-09	1.21E-24	/	/	/	/
MELO3C005129.2	ABA2, Xanthoxin dehydrogenase	/	/	/	-3.0656	-1.2975	1.8851	/	/	/	1.03E-37	0.002245	2.12E-14	/	/	/	1.79E-36	0.004023	1.66E-12
MELO3C002744.2	NCED2,9-cis-epoxycarotenoid dioxygenase	-4.5162	-6.8918	/	-4.8066	/	/	4.40E-14	1.77E-10	/	6.02E-15	/		6.05E-13	5.90E-10	/	3.24E-14	/	/
MELO3C007127.2	NCED2, 9-cis-epoxycarotenoid dioxygenase	-2.8561	1.9792	5.9041	3.8600	6.8789	/	9.94E-13	2.67E-18	4.00E82	1.09E-40	1.47E-85	/	1.20E-11	1.40E-17	2.22E-80	2.15E-39	7.56E-84	/
MELO3C023086.2	NCED6, 9-cis-epoxycarotenoid dioxygenase	1.2511	2.57400	1.5643	/	/	/	7.16E-13	2.99E-70	1.04E-20	/	/	/	8.76E-12	7.88E-69	8.13E-20	/	/	/

Table S7 Screened list of differentially expressed genes related to chlorophyll metabolism

Gene ID	Description	Gene expression					
		1_6	3_6	4_6	1_14	3_14	4_14
MELO3C004867.2	PAO, Pheophorbide a oxygenase	45.6706	20.7863	46.4835	19.5245	26.5158	34.0857
MELO3C035541.2	PAO, Pheophorbide a oxygenase	0.8315	0.1361	1.2249	1.5829	3.1732	3.7297
MELO3C005189.2	POX1, Protoporphyrinogen oxidase 1	9.1998	20.1105	24.5975	9.1281	18.9724	21.9347
MELO3C008208.2	POX2, Protoporphyrinogen oxidase 2	6.0428	5.7562	7.6904	16.3070	7.6519	8.7947
MELO3C006242.2	COX10, Protoheme IX farnesyltransferase	30.9642	19.7465	25.1298	33.9238	19.1044	22.8777
MELO3C016120.2	COX15, Cytochrome c oxidase assembly protein	4.5666	6.7467	9.6766	7.3530	8.1418	9.3589
MELO3C007233.2	CHLI, Magnesium-chelatase subunit ChlI	32.7924	57.5428	127.1322	25.2638	93.1005	53.4685
MELO3C014571.2	CHLD, Magnesium-chelatase subunit ChlD	10.1434	15.9460	12.8227	16.8399	14.4142	7.1206
MELO3C026000.2	CHLG, Chlorophyll synthase	34.9361	44.1191	33.9946	19.6196	41.1236	30.9775
MELO3C026160.2	CHLM, Magnesium protoporphyrin IX methyltransferase	63.2184	25.0710	41.0206	20.2988	31.7037	26.4776
MELO3C014712.2	CHLP, Geranylgeranyl diphosphate reductase	0.6132	0.4088	2.5394	0.8210	0.6142	2.1636
MELO3C017176.2	CHLP, Geranylgeranyl diphosphate reductase	23.2812	68.1179	127.8698	23.4735	96.3832	30.7724
MELO3C023131.2	CHLH, Magnesium-chelatase subunit ChlH	5.5349	68.0900	145.0748	1.2678	52.6180	58.8092
MELO3C014286.2	CLH2, Chlorophyllase-2	4.4229	8.2237	11.6441	6.1961	10.2281	12.2702
MELO3C011113.2	HEMA1, Glutamyl-tRNA reductase 1	33.9059	45.5292	51.8491	23.8664	51.2897	20.1317
MELO3C018565.2	HEMA2, Glutamyl-tRNA reductase 2	142.9519	22.7445	5.0638	53.4152	14.6574	12.1401
MELO3C008086.2	HEMB1, Delta-aminolevulinic acid dehydratase 1,	78.8422	64.7055	75.5363	54.2364	70.8675	63.0163
MELO3C024010.2	HEMC, Porphobilinogen deaminase	18.8412	25.2184	81.1137	22.5093	42.6499	31.8089
MELO3C016131.2	HEME1, Uroporphyrinogen decarboxylase 1	13.5793	17.1684	26.7788	22.4659	19.5783	18.5686
MELO3C010762.2	HEMH, Ferrochelatase-2	72.0768	24.2509	31.9115	36.2752	20.0622	37.5318
MELO3C013592.2	PORA, Protochlorophyllide reductase	3.2375	1.1783	8.4173	1.8176	3.4127	2.5397
MELO3C016714.2	PORA, Protochlorophyllide reductase	148.3747	339.2976	571.7425	105.1112	417.2457	214.6357
MELO3C006358.2	HCAR, 7-hydroxymethyl chlorophyll a reductase	4.8837	15.6582	25.3445	4.2049	21.5738	17.0589
MELO3C010350.2	HO1, Heme oxygenase 1	11.2102	21.6180	13.0663	13.1509	22.1563	16.8812
MELO3C016736.2	HO2, Probable inactive heme oxygenase 2	3.8183	6.2914	6.3906	4.3832	6.1334	5.9387
MELO3C029500.2	HY2, Phytochromobilin ferredoxin oxidoreductase	8.4997	8.0730	6.4240	4.5886	12.2069	9.7443
MELO3C010614.2	CAO, Chlorophyllide a oxygenase	16.7366	89.2360	99.4100	17.3610	88.0594	60.4608
MELO3C026802.2	CRD1, Magnesium-protoporphyrin IX monomethyl ester [oxidative] cyclase	73.2959	253.0168	490.1623	34.7762	322.8260	164.4282
MELO3C006606.2	DVR, Divinyl chlorophyllide 8-vinyl-reductase	4.0426	5.4728	5.6100	6.5743	6.4370	2.3953
MELO3C010892.2	UROS, Uroporphyrinogen-III synthase	11.2061	8.2009	17.0411	9.8555	8.7166	11.3158
MELO3C015227.2	At5g26710, Glutamate--tRNA ligase	264.1536	28.5980	40.2471	295.1378	31.4403	39.5561
MELO3C015693.2	OVA3, Glutamate--tRNA ligase	17.0231	24.8484	32.3586	18.3372	26.8148	26.5627
MELO3C019619.2	Os05g0361200, Ferrochelatase-2	43.3270	50.3943	35.7526	32.1812	43.3126	28.7863
MELO3C016226.2	CPX, Oxygen-dependent coproporphyrinogen-III oxidase	187.4576	46.2469	70.3640	239.4888	48.0828	43.0569
MELO3C018572.2	GSA, Glutamate-1-semialdehyde 2,1-aminomutase	62.8088	53.8178	63.1913	70.8763	55.8806	59.8852
MELO3C025347.2	NYC1, Probable chlorophyll(ide) b reductase	13.7839	140.9867	131.0567	10.3011	211.3061	247.3177
MELO3C019918.2	NOL, Chlorophyll(ide) b reductase NOL	4.3169	8.5939	11.4912	6.6063	8.7110	12.7130
MELO3C020115.2	DCUP, Uroporphyrinogen decarboxylase	28.0328	33.1030	42.0972	27.9404	38.3389	22.7795
MELO3C020912.2	RCCR, Red chlorophyll catabolite reductase	61.4929	72.7201	85.3020	68.1077	84.1772	44.0519
MELO3C020913.2	RCCR, Red chlorophyll catabolite reductase	32.4496	35.8398	84.5843	84.4801	32.2111	40.4482

MELO3C013522.2	FH, Frataxin	15.8330	17.5373	22.7346	18.0707	20.8974	28.9511

Table S8 List of differentially expressed genes related to chlorophyll metabolism (ko00860).

Gene ID	Description	log2 Fold change		
		1_6 vs. 4_6	1_14 vs. 4_14	4_14 vs. 4_6
MELO3C005189.2	POX1, Protoporphyrinogen oxidase 1	1.2044	1.1582	/
MELO3C007233.2	CHLI, Magnesium-chelatase subunit ChlI	1.7529	/	-1.3026
MELO3C014712.2	CHLP, Geranylgeranyl diphosphate reductase	1.8435	1.2911	/
MELO3C017176.2	CHLP, Geranylgeranyl diphosphate reductase	2.2565	/	-2.1035
MELO3C023131.2	CHLH, Magnesium-chelatase subunit ChlH	4.5112	5.4357	-1.3508
MELO3C002961.2	CHLH1, Magnesium-chelatase subunit ChlH1	/	-5.9663	/
MELO3C014286.2	CLH2, Chlorophyllase-2	1.1656	/	/
MELO3C014571.2	CHLD, Magnesium-chelatase subunit ChlD	/	-1.3461	/
MELO3C018565.2	HEMA2, Glutamyl-tRNA reductase 2	-5.0718	-2.2313	/
MELO3C024010.2	HEMC, Porphobilinogen deaminase	1.8903	/	-1.3969
MELO3C010762.2	HEMH, Ferrochelatase-2	-1.4186	/	/
MELO3C013592.2	PORA, Protochlorophyllide reductase	1.1457	/	-1.7788
MELO3C016714.2	PORA, Protochlorophyllide reductase	1.7461	/	-1.4648
MELO3C006358.2	HCAR, 7-hydroxymethyl chlorophyll a reductase	2.1803	1.9189	/
MELO3C010614.2	CAO, Chlorophyllide a oxygenase	2.3498	1.6968	/
MELO3C026802.2	CRD1, Magnesium-protoporphyrin IX monomethyl ester [oxidative] cyclase	2.5334	2.1383	-1.6295
MELO3C015227.2	At5g26710, Glutamate--tRNA ligase	-2.9624	-3.0034	/
MELO3C016226.2	CPX, Oxygen-dependent coproporphyrinogen-III oxidase	-1.6559	-2.5769	/
MELO3C025347.2	NYC1, Probable chlorophyll(ide) b reductase	3.0265	4.4819	/
MELO3C019918.2	NOL, Chlorophyll(ide) b reductase NOL	1.1909	/	/
MELO3C020912.2	RCCR, Red chlorophyll catabolite reductase	1.1555	/	-1.0058
MELO3C020913.2	RCCR, Red chlorophyll catabolite reductase	/	-1.1696	-1.1170
MELO3C006606.2	DVR, Divinyl chlorophyllide 8-vinyl-reductase	/	-1.5634	-1.2839
MELO3C011113.2	HEMA1, Glutamyl-tRNA reductase 1	/	/	-1.4143
MELO3C035541.2	PAO, Pheophorbide a oxygenase	/	/	1.5454
MELO3C035546.2	PAO, Pheophorbide a oxygenase	/	/	1.8917
